# The influence on oxidative stress markers, inflammatory factors and intestinal injury-related molecules in Wahui pigeon induced by lipopolysaccharide

**DOI:** 10.1371/journal.pone.0251462

**Published:** 2021-05-12

**Authors:** Fei Wang, Jin Liu, Xiaofen Hu, Youbao Zhong, Feng Wen, Xiaoen Tang, Shanshan Yang, Shengwei Zhong, Zuohong Zhou, Xu Yuan, Yong Li

**Affiliations:** 1 College of Animal Science and Technology, Jiangxi Agricultural University, Nanchang, 330045, Jiangxi, China; 2 Technology Center of Experimental Animals, Jiangxi University of Traditional Chinese Medicine, Nanchang, 330004, Jiangxi, China; 3 College of Life Science and Engineering, Foshan University, Foshan, 528231, Guangdong, China; 4 Fuzhou Husbandry Breeding Farm, Linchuan, 344000, Jiangxi, China; University of Messina, ITALY

## Abstract

**Introduction:**

The intestinal structure is the foundation for various activities and functions in poultry. An important question concerns the changes in the intestinal status under endotoxin stimulation. This study aimed to investigate the mechanism of intestinal injury induced by lipopolysaccharide (LPS) in Wahui pigeons.

**Methods:**

Thirty-six 28-day-old healthy Wahui pigeons were randomly divided into two groups. The experimental group was injected with LPS (100 μg/kg) once per day for five days, and the control group was treated with the same amount of sterile saline. Blood and the ileum were collected from pigeons on the first, third, and fifth days of the experiment and used for oxidative stress assessment, inflammatory factor detection, histopathological examination, and positive cell localization. In addition, intestinal injury indices and mRNA expression levels (tight junction proteins, inflammatory cytokines, and factors related to autophagy and apoptosis) were evaluated.

**Results:**

Villi in the ileum were shorter in the LPS group than in the control group, and D-lactic acid levels in the serum were significantly increased. Glutathione and catalase levels significantly decreased, but the malondialdehyde content in the serum increased. TNF-α and IL-10 were detected at higher levels in the serum, with stronger positive signals and higher mRNA expression levels, in the LPS group than in the control group. In addition, the levels of TLR4, MyD88, NF-κB, and HMGB1 in the inflammatory signaling pathway were also upregulated. Finally, the mRNA expression of Claudin3, Occludin, and ZO-1 was significantly decreased; however, that of Beclin1 and Atg5 was increased in the LPS group.

**Conclusion:**

Ileal pathological changes and oxidative stress were caused by LPS challenge; it is proposed that this triggering regulates the inflammatory response, causing excessive autophagy and apoptosis, promoting intestinal permeability, and leading to intestinal injury in Wahui pigeons.

## 1. Introduction

It is well known that poultry meat is very popular owing to its competitive price, lack of religious and geographical restrictions, and high nutritional value [1]. Pigeon meat contains a high protein and low fat content, and its digestion and absorption rate can reach more than 91%. However, in pigeons bred for meat, salmonellosis frequently occurs. This disease can lead to fever, dysentery, arthritis, neurological symptoms, or acute septicemia in later stages, with high mortality in squabs. LPS, which is a known endotoxin, is a key molecule in the outer membrane of gram-negative bacteria, and is considered a main pathogenic factor [2]. During endotoxemic shock, immune cells release several mediators and cytokines that lead to hypotension, fever and tissue injury [3]. Stimulation with LPS has extensively been employed in many endotoxic models for the understanding of underlying complex molecular mechanisms of endotoxin-mediated acute intestine tissue damage [4–7].

LPS can cause oxidative stress by promoting the formation of reactive oxygen species (ROS) in several cell types [4,8], indicating that oxidative stress plays an important role in the pathogenesis of LPS. Oxidative stress induced by ROS is closely related to inflammatory responses [9]. High LPS exposure can increase the production of ROS and take a regulatory role in inflammation, leading to peripheral inflammation in many models [10]. Excessive ROS can cause oxidative stress, autophagy, and apoptosis in hepatocytes [11,12]. In addition, LPS can promote epithelial cell apoptosis and proliferation and induce proinflammatory processes that cause inflammatory responses through the nuclear factor-κB (NF-κB) signaling pathway [13].

Inflammation is a protective reaction to infections and tissue damage that is triggered by innate immunocytes responding to external stimuli [14]. Immune cells can initiate signaling cascades that activate crucial transcription factors, including NF-κB, mitogen-activated protein kinases (MAPKs), and activator protein 1, which in turn regulate inflammation-specific genes [15,16]. The interaction between LPS and Toll-like receptor 4 (TLR4) leads to the formation of an LPS signaling complex consisting of surface molecules, including myeloid differentiation primary response gene 88 (MyD88), toll-interleukin-1 receptor domain-containing adapter inducing interferon β, and TNF-α receptor association factor 6, and the activation of transcription factors, which then induce the activation of the inflammatory response [17].

The intestinal barrier is considered to be a selective barrier against exogenous noxious antigens and pathogens [18]. Disruption of the intestinal barrier promotes the transit of luminal antigens to the subepithelial tissues, inducing mucosal and systemic inflammatory responses, which is the major pathogenesis in intestinal disease [19]. Multiple factors, including inflammation and oxidative stress, can cause intestinal barrier damage [18–21]. Evidence has demonstrated that LPS stimulates intestinal immune cells to rapidly produce proinflammatory cytokines, which leads to structural and functional injury in the intestine [22]. Furthermore, exposure to intraperitoneal LPS at low doses to laboratory animals can selectively increase intestinal permeability by tight junction modulation and cause a pro-inflammatory state in the intestine [23]. The mucosal immune system is well known as an important part of the entire immune network in poultry. As an independent immune system, it plays critical roles with unique structure-function features in fighting against infections [24]. Intestinal mucosa is not only the crucial site for food digestion and nutrient absorption but also an immune structure, where the largest number of immune cells collectively presents to form a strict defense system [25]. Therefore, normal intestinal structure plays a key role for its immunity. The current study explored the dynamic changes in the intestinal microstructure, degree of oxidative stress, expression of inflammatory factors, and activity of autophagy, apoptosis in Wahui pigeons induced by *Salmonella* LPS. The objective was to clarify the pathogenic mechanism of intestinal injury in pigeons with LPS treatment and to provide theoretical evidence for intestinal immunity.

## 2. Materials and methods

### Experimental animals

In this experiment, thirty-six 28-day-old healthy Wahui pigeons (body weight: 0.47 ± 0.05 kg) were purchased from Pigeon Farm (Nanchang, Jiangxi, China) and had *ad libitum* access to feed and water. After two days of pre-feeding to alleviate any stress response, the pigeons were randomly divided into two groups. The LPS group was injected intraperitoneally with LPS (L6511; Sigma-Aldrich, St. Louis, MO, USA) at 100 μg/kg body weight every day, and the control group was injected with the same volume of saline. On the first, third, and fifth days of the experiment, six pigeons were randomly selected from each group for blood collection (5 mL) after a anaesthetization by CO2 inhalation. Subsequently, we dissected the abdominal cavity and instantly harvested the ileum samples. Parts of the ileum was fixed in Bouin’s solution and 4% paraformaldehyde for 24–72 h, and the remaining portion was quickly placed in liquid nitrogen and then stored in a freezer at -80°C. All procedures in this experiment were approved by the Institutional Animal Care and Use Committee of Jiangxi Agricultural University (approval number: JXAULL-2020-35).

### Detection of biochemical and antioxidant indices in the serum

The main compounds of interest in the serum were superoxide dismutase (SOD), catalase (CAT), glutathione (GSH), and malondialdehyde (MDA), which were evaluated by using a spectrophotometric method according to the corresponding diagnostic kits (Nanjing Jiancheng Bioengineering Institute Inc., Nanjing, Jiangsu, China). The content of D-lactic acid (D-LA) in the serum was measured using enzyme-linked immunosorbent assay (ELISA). The method was carried out according to the instructions for the serum ELISA kit (Nanjing Jiancheng Bioengineering Institute Inc.).

### Detection of inflammatory factors in the serum

HMGB1, TNF-α, and IL-10 concentrations in the serum were measured by ELISA according to the corresponding kit instructions (Nanjing Jiancheng Bioengineering Institute Inc.). The concentrations of HMGB1, TNF-α, and IL-10 were calculated according to a standard curve and are expressed as ng/L.

### Hematoxylin-eosin (HE) staining

Fixed tissues were rinsed with running water for 5–10 h and then dehydrated in 70%, 80%, 90%, 95%, and anhydrous ethanol using a LEICA ASP200S automatic dehydrator (LEICA Camera AG, Wetzlar, Germany). Samples were then rinsed in xylene three times and dipped in paraffin for 3–4 h. Then, 4-μm-thick sections were made with a LEICA semiautomatic microtome (LEICA Camera AG). Half of the sections were processed by HE staining, and the other half was used for immunohistochemical staining, as previously described [26]. The tissue sections were sequentially subjected to two rinses in xylene and two rinses in anhydrous ethanol, 95%, 90%, 80%, and 70% ethanol and then stained in a 1% hematoxylin solution for 1 min. Subsequently, the sections were rapidly processed through a color separation solution and blue-black fluid and dyed in an eosin solution for 30–45 s. Finally, the sections were dehydrated in 70%, 80%, 90%, 95%, and anhydrous ethanol; rinsed twice in xylene; and then mounted with neutral balsam and cover slips. The intestinal microstructure was observed and imaged using an Olympus BX53 microscope (Olympus, Japan), and ten villus heights (VH) and ten crypt depths (CD) were measured in each visual field. Ten visual fields were evaluated in each section at random, and the ratio of villus height/crypt depth (VH/CD) was calculated.

### Immunohistochemical staining

Immunohistochemistry was performed as previously described [27]. After deparaffinization, an endogenous peroxidase blocking solution was added to tissue sections, which were then incubated at 37°C for 15 min. Antigen retrieval was performed at 98°C for 12 min, and then the sections were incubated with goat serum at 37°C for 20 min. Rabbit anti-TNF-α (1:600) and mouse anti-IL-10 (1:250) polyclonal antibodies were added dropwise to the sections and incubated at 4°C overnight. After the primary antibody was ligated, the sections were incubated with biotinylated goat anti-rabbit and goat anti-mouse secondary antibodies at 37°C for 30 min, combined with peroxidase-labeled streptavidin at room temperature for 10 min, and then visualized using a DAB coloring agent for 1–5 min. The sections were counterstained in a hematoxylin solution for 2 min, processed in 1 mL/L hydrochloric acid alcohol differentiation and blue-black fluid, and rinsed with distilled water. Finally, the sections were dehydrated, cleared and mounted with neutral balsam and cover slips. The primary antibody was replaced with a 0.01 mol/L PBS solution in the control group, but the remaining steps were performed as described above. All sections were observed and imaged using an Olympus BX53 microscope (Olympus, Japan), and six fields of vision were chosen according to different regions of each ileum section. Image-Pro Plus (IPP) 6.0 software (Media Cybernetics, USA) was used to calculate the integral optical density (IOD) for positive staining and the graphs were prepared by Prism software version 8.0 (GraphPad Software, Inc., San Diego, USA). The average optical density (AOD = IOD/area) was calculated.

### RNA extraction and cDNA synthesis

RNA was extracted from the ileum using a TRIzol extraction method. Briefly, frozen specimens were powdered in liquid nitrogen and homogenized in RNAiso Plus (TAKARA Bio Inc., Shiga, Japan); then, 500 mL of sample was transferred to a tube. Chloroform (200 μL) was added to each tube, and the tubes were violently shaken for 15 s and centrifuged at 4°C and 12000 rpm for 15 min. Each sample was divided into three layers: the yellow organic phase, middle layer, and upper colorless water phase. The upper water phase was transferred to a new 1.5-mL tube, and then 500 μL of isopropanol was added to this tube and mixed gently. The mixture was kept at room temperature for 10 min and then centrifuged at 4°C and 12000 rpm for 10 min. After the supernatant was discarded, the isolated total RNA was washed with 1 mL of 95% ethanol prepared with RNase-free double-distilled water. Then, 30 μL of diethylpyrocarbonate water (TAKARA Bio Inc.) was added to fully dissolve the precipitate, which was then placed in a freezer at -80°C (Thermo Fisher Scientific, Waltham, MA, USA). RNA concentration and optical density values were detected using an ultraviolet spectrophotometer (Beckman Coulter Inc., Brea, CA, USA). First-strand cDNA was synthesized using the EasyScript One-Step gDNA Removal and cDNA Synthesis SuperMix Kit (TransGen Biotech Co., Ltd., Beijing, China) and then placed in a freezer at -20°C.

### Quantitative real-time PCR detection

The mRNA expression of related genes in the ileum was detected by real-time fluorescence quantitative PCR. The cDNA generated from each tissue sample was used as a template, and the *GAPDH* gene was used as an internal reference. The reaction system had a total volume of 20 μL and consisted of the following components: 10 μL of 2 × qPCR Mix, 0.4 μL of 10 μmol/L upstream and downstream primers (S1 Table), 2 μL of cDNA, and 7.2 μL of nuclease-free water. The PCR conditions were as follows: predenaturation of the template at 94°C for 30 s and a total of 42 cycles of 94°C for 5 s and 62°C for 30 s for template amplification, with a final slow increase in temperature by 0.5°C every 10 s from 55°C to 95°C. Results were calculated using the 2^-△△Ct^ method.

### Statistical analysis

One-way analysis of variance was performed to compare differences between the control and LPS groups using SPSS 17.0 software (Chicago, IL, USA), and differences were considered significant if p < 0.05 (*p < 0.05, **p < 0.01). All data are presented as the mean ± standard deviation. Graph Prism 8.0 software (GraphPad, San Diego, CA, USA) was used to generate the corresponding graphs.

## 3. Results

### Effects of LPS on antioxidant indices

**Fig 1:**
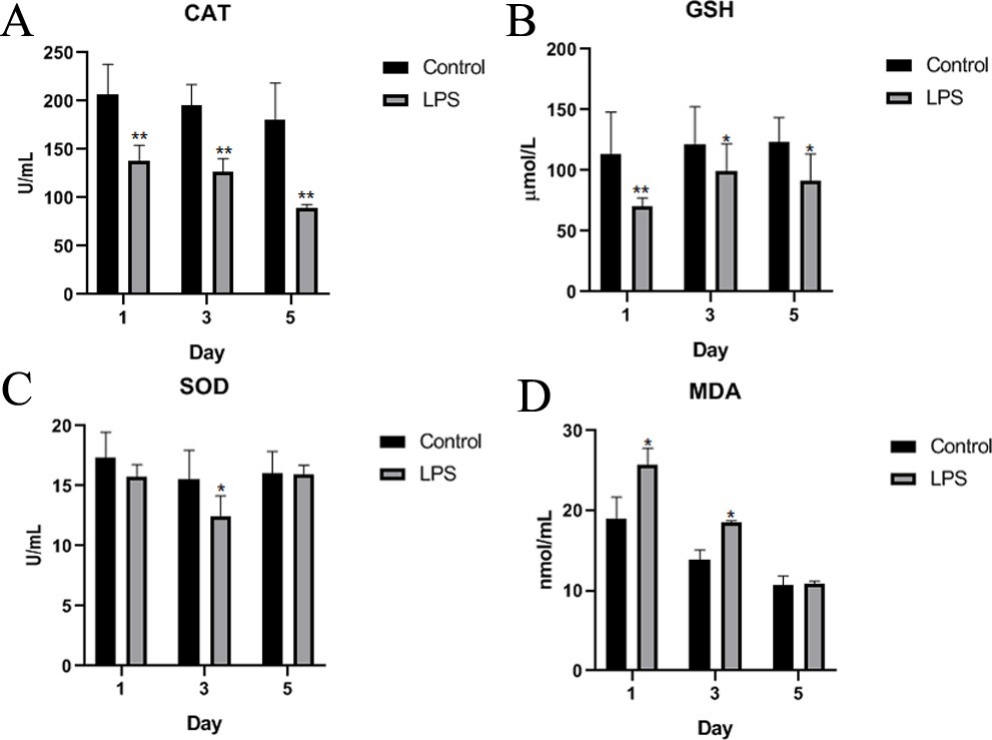
Antioxidant indices of Wahui pigeons treated with LPS or saline. The antioxidant indices of Wahui pigeons were detected in both groups on the first, third, and fifth days. Data represent the mean ± standard deviation (n = 6 in each group); *p < 0.05, **p < 0.01. Abbreviations: CAT—catalase; GSH—glutathione; SOD—superoxide dismutase; MDA—malondialdehyde.

Oxidative stress is a critical factor involved in the barrier disruption in intestinal diseases [28]. It can destroy essential cellular molecules, such as lipids, proteins, and DNA, resulting in a series of diseases, including intestinal barrier injury [20]. Therefore, several antioxidant indices were tested for evaluating oxidative stress. Compared with those in the control group, the activity of catalase (CAT) and content of glutathione (GSH) in the LPS group were significantly decreased in the serum on the first, third, and fifth days, while the malondialdehyde (MDA) content was increased on the first and third days (Fig 1A, 1B and 1D). Superoxide dismutase (SOD) showed lower activity in the serum of LPS-treated Wahui pigeons than in that of saline-treated Wahui pigeons (Fig 1C).

### Effects of LPS on inflammatory factors

**Fig 2:**
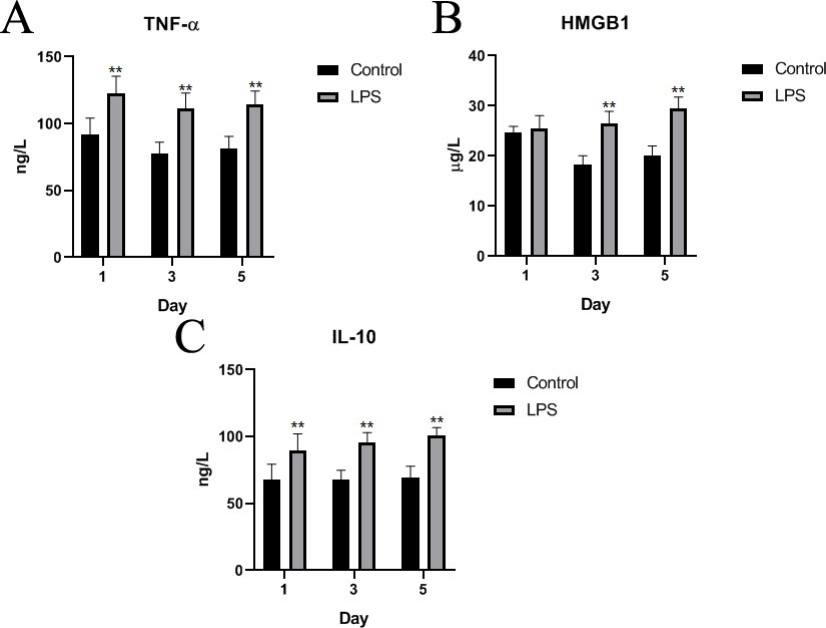
TNF-α, HMGB1, and IL-10 concentrations detected in the serum by ELISA on the first, third and fifth days. Data represent the mean ± standard deviation (n = 6 in each group); *p < 0.05, **p < 0.01.

According to enzyme-linked immunosorbent assay (ELISA) results (Fig 2), the concentrations of TNF-α and IL-10 were significantly greater in LPS-treated pigeons than in saline-treated pigeons on day 1 (p < 0.01), while the concentration of HMGB1 showed no significant difference between the two groups (p > 0.05). The TNF-α, HMGB1, and IL-10 concentrations were extremely significantly greater in LPS-treated Wahui pigeons than in saline-treated pigeons on days 3 and 5 (p < 0.01).

### Effects of LPS on intestinal function and morphology

**Fig 3:**
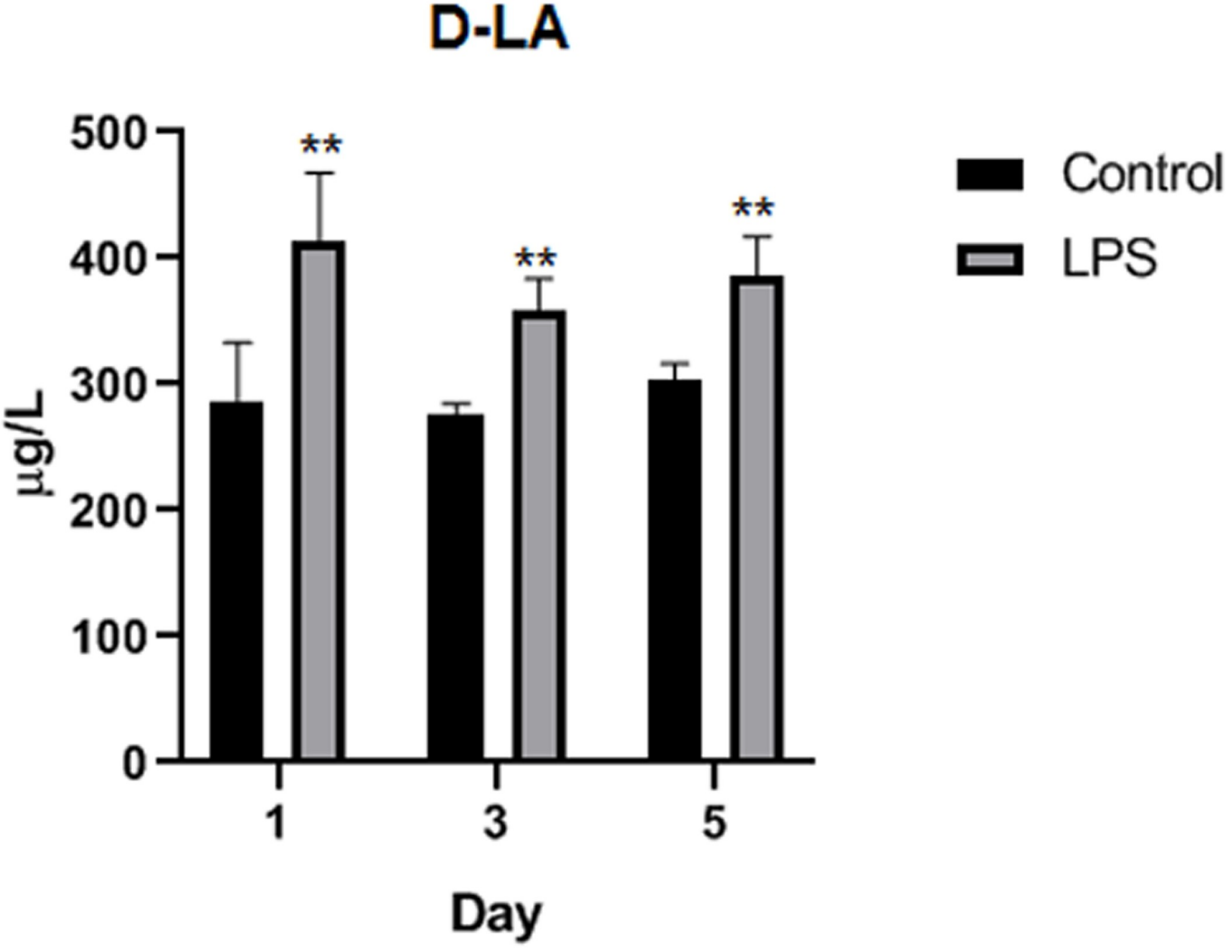
Detection of D-LA content in serum of Wahui pigeons. Data represent the mean ± standard deviation (n = 6 in each group); *p < 0.05, **p < 0.01.

D-LA is a chemical marker that is usually present at low levels in the circulatory system of healthy individuals; these levels increase significantly with the destruction of the intestinal barrier [29]. After LPS stimulation, on the first, third, and fifth days, D-LA levels were significantly higher in the LPS-treated pigeons than in the saline-treated pigeons evaluated on the same date (p < 0.01 for all three days; Fig 3).

**Fig 4:**
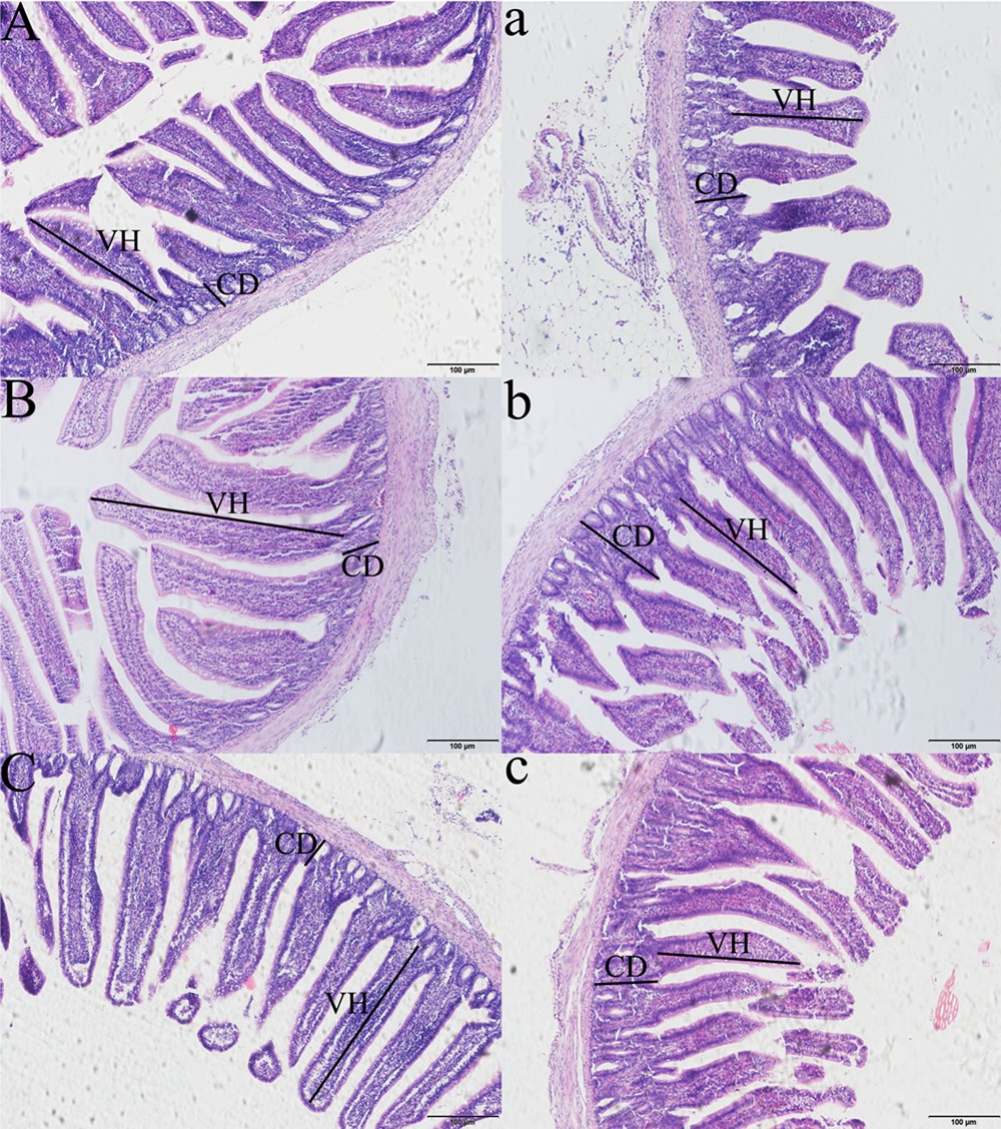
The intestinal microstructure of Wahui pigeons after LPS or saline treatment (100×). **A**, **B,** and **C** show the ileum of saline-treated Wahui pigeons on days 1, 3, and 5, respectively; **a**, **b**, and **c** show the ileum of LPS-treated Wahui pigeons on days 1, 3, and 5, respectively.

Compared with pigeons of the same age injected with saline, pigeons treated with LPS showed significant shortening of the ileal villi, deeper crypts (p < 0.05, S2 Table and Fig 4), and a significantly decreased ratio of villus height/crypt depth (VH/CD) (p < 0.01, S2 Table and Fig 4).

### Localization of TNF-α and IL-10 in the intestine

**Fig 5:**
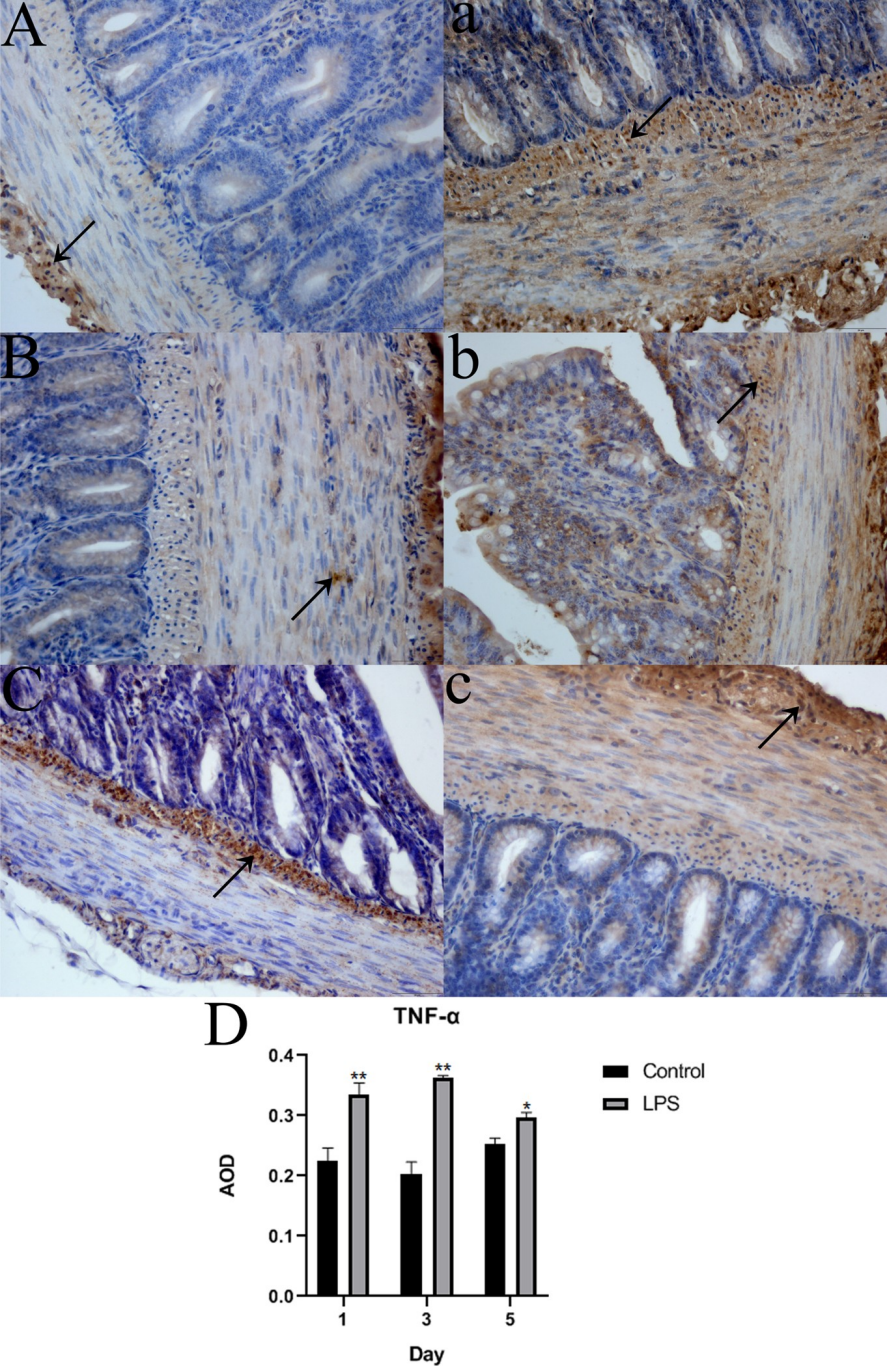
Detection of TNF-α in the ileum of LPS- and saline-treated Wahui pigeons. The black arrows indicate TNF-α-positive cells (400**×)**. **A**, **B**, and **C** show the ileum of saline-treated Wahui pigeons on days 1, 3, and 5, respectively; **a**, **b**, and **c** show the ileum of LPS-treated Wahui pigeons on days 1, 3, 5, respectively. **D**: The average optical density (AOD) of TNF-α. Data represent the mean ± standard deviation (n = 6 in each group), and six visual fields were evaluated in each section; *p < 0.05, **p < 0.01.

**Fig 6:**
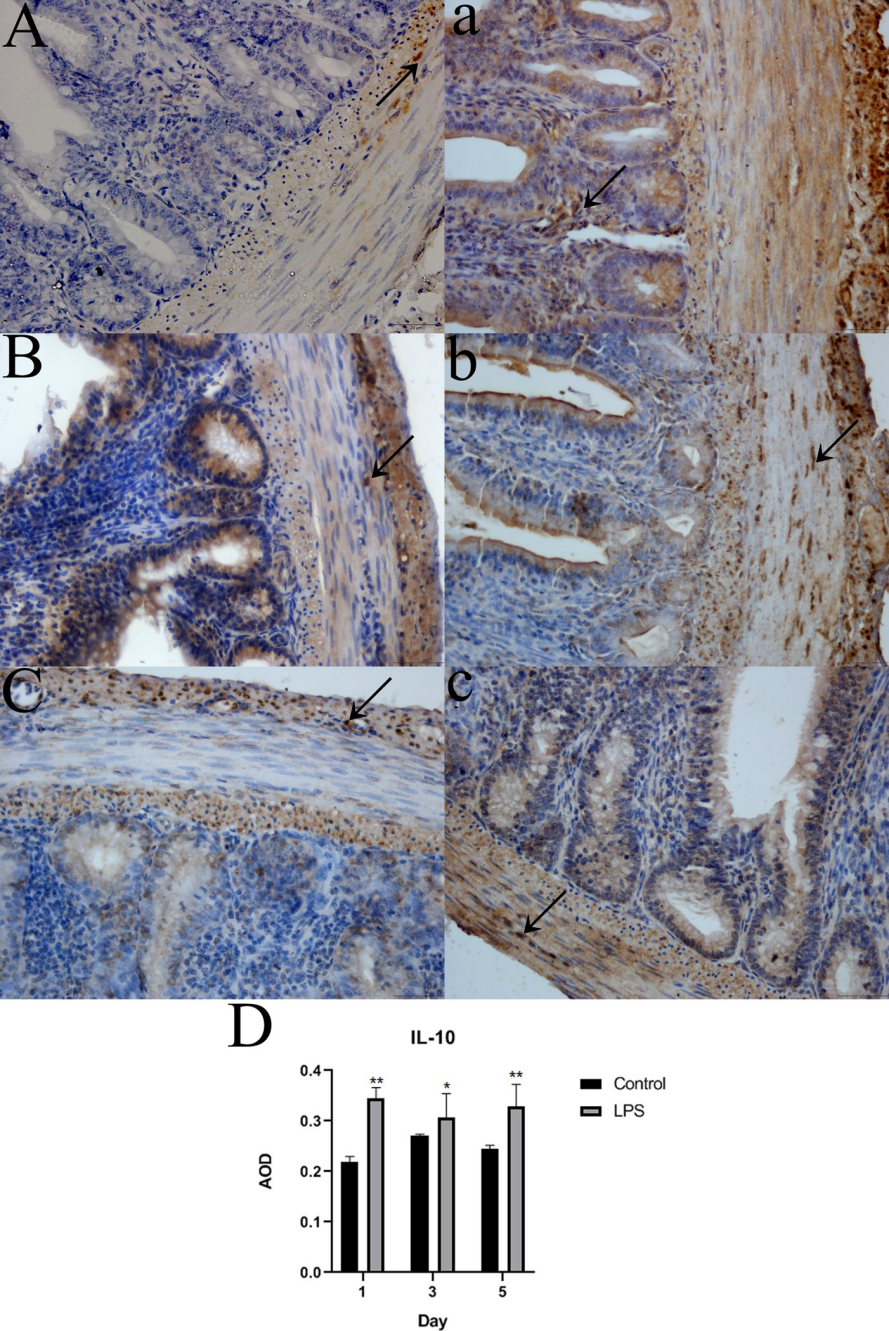
Detection of IL-10 in the ileum of LPS- and saline-treated Wahui pigeons. The black arrows indicate IL-10-positive cells (400×). **A**, **B**, and **C** show the ileum of saline-treated Wahui pigeons on days 1, 3, and 5, respectively; **a**, **b**, and **c** show the ileum of LPS-treated Wahui pigeons on days 1, 3, and 5, respectively. **D**: The average optical density (AOD) of IL-10. Data represent the mean ± standard deviation (n = 6 in each group), and six visual fields were evaluated in each section; *p < 0.05, **p < 0.01.

Positive immunohistochemical staining for TNF-α and IL-10 were predominantly detected in the cytoplasm, as indicated by brown staining. TNF-α and IL-10 were diffusely distributed in the villi, crypt, muscularis mucosa, muscular layer, and serosa and were predominant in the muscularis mucosa, muscular layer, and serosa. Significantly more positive areas of TNF-α and IL-10 staining were found in the intestine of LPS-treated Wahui pigeons than in that of saline-treated Wahui pigeons (Figs 5 and 6). The average optical density (AOD) was significantly different between the two groups on different days. The AOD of the TNF-α-positive cells was significantly higher in the LPS-treated Wahui pigeons than in the saline-treated pigeons on days 1, 3, and 5 (p < 0.01 for all three days; Fig 5D). The AOD of the IL-10-positive cells was higher in the LPS-treated Wahui pigeons than in the saline-treated pigeons on days 1, 3 and 5 (p < 0.01, p < 0.05, and p < 0.01, respectively; Fig 6D).

### Occludin, Claudin3, and ZO-1 gene assays at intestinal tight junctions

**Fig 7:**
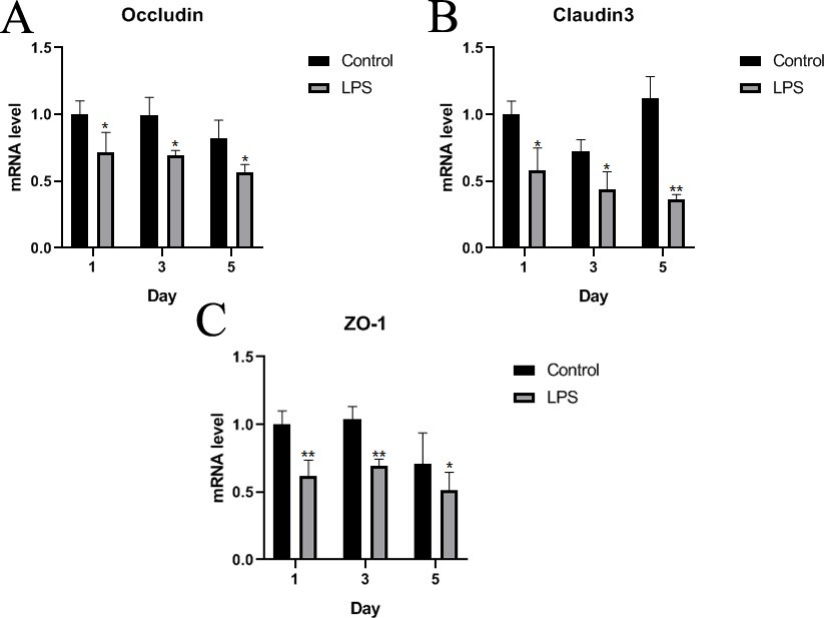
Evaluation of the intestinal tight junctions of Wahui pigeons treated with LPS or saline. The mRNA expression of Occludin, Claudin3, and ZO-1 in the ileum was detected by RT-qPCR on the first, third, and fifth days after LPS or saline treatment, and *GAPDH* was used as a reference gene. Data represent the mean ± standard deviation (n = 6 in each group); For Wahui pigeons of the same age, *p < 0.05 and **p < 0.01.

As essential cell-cell junction for forming intestinal barrier, tight junction takes a role in structural strength and stability of intestine, the levels of gene expression, including Occludin, Claudin3, and ZO-1 related to tight junction, were detected. Compared to those in the control group, the expression levels of Occludin, Claudin3, and ZO-1 in the LPS group were significantly decreased on days 1, 3, and 5 (Fig 7). In addition, the expression levels of Occludin and Claudin3 in the LPS group showed a downward trend during the experiment (Fig 7A and 7B).

### Expression changes of key genes in multiple signaling pathways induced by LPS

**Fig 8:**
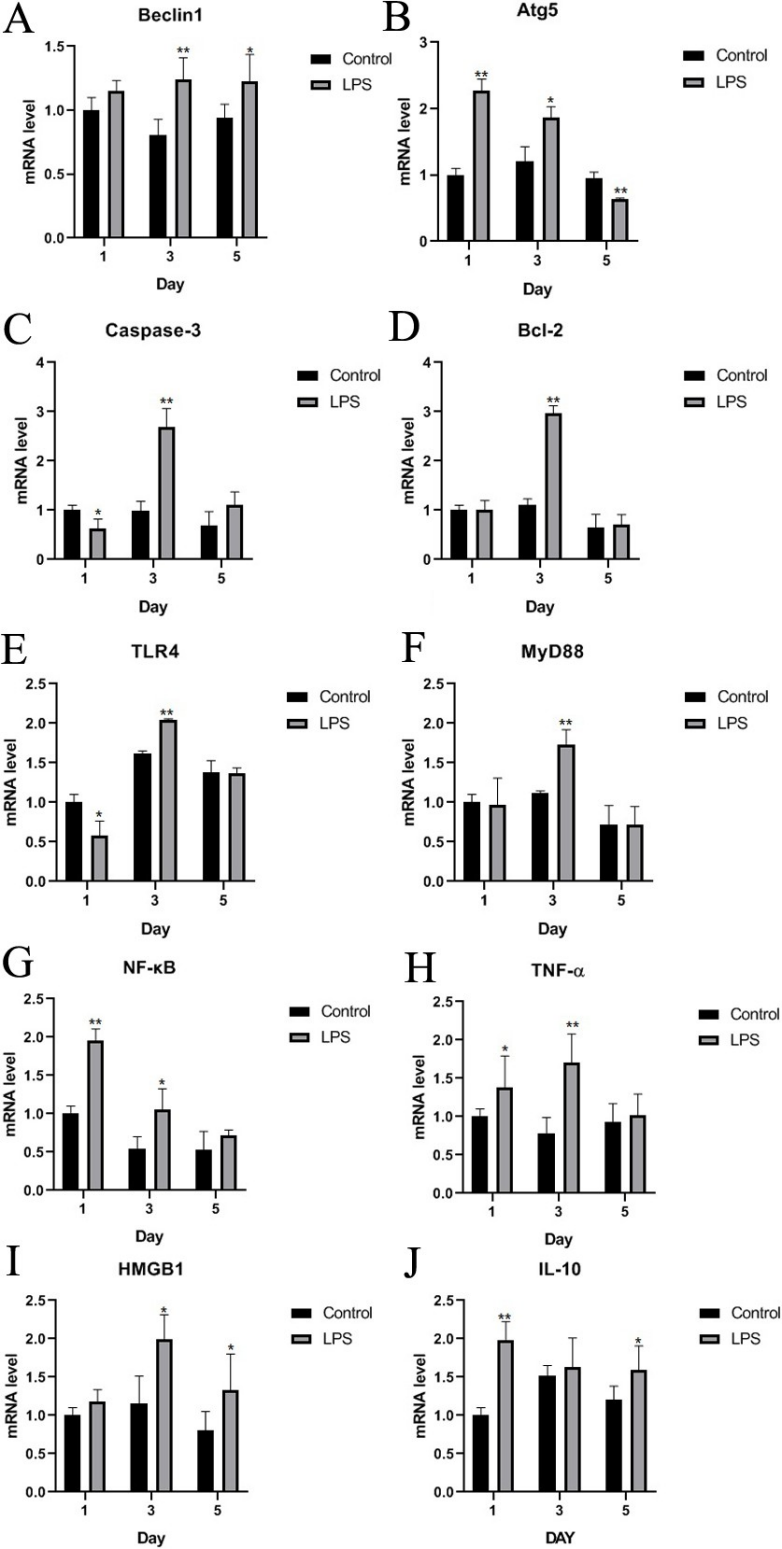
Levels of indicators of various signaling pathways in Wahui pigeons treated with LPS or saline. The mRNA expression levels of Beclin1, Atg5, Caspase-3, Bcl-2, TLR4, MyD88, NF-κB, TNF-α, HMGB1, and IL-10 were detected by RT-qPCR on the first, third, and fifth days after LPS or saline treatment, and *GAPDH* was used as a reference gene. Data represent the mean ± standard deviation (n = 6 in each group); For Wahui pigeons of the same age, *p < 0.05 and **p < 0.01.

In the autophagic signaling pathway, both factors showed similar results. *Atg5* expression in the LPS group was significantly higher than that in the control group on days 1, 3, and 5 (p < 0.01, p < 0.05, p < 0.01, respectively; Fig 8B), and Beclin1 expression was also significantly higher in the LPS group than in the control group on days 3 and 5 (p < 0.01 and p < 0.05, respectively), but the difference was not significant on day 1 (Fig 8A).

In the apoptotic signaling pathway, the expression level of Caspase-3 was significantly lower in the LPS group than in the control group on day 1 (p < 0.05); however, the expression levels of Caspase-3 and Bcl-2 were significantly increased on day 3 (p < 0.01 and p < 0.01, respectively) and were also elevated on day 5 (p > 0.05, Fig 8C and 8D).

In the inflammatory signaling pathway, TLR4 was expressed at a significantly lower level in the LPS group than in the control group (p < 0.05), while NF-κB expression was significantly higher on day 1 (p < 0.01). The expression of TLR4, MyD88, and NF-κB in the LPS-treated Wahui pigeons was significantly higher than that in the saline-treated pigeons on day 3 (p < 0.01, p < 0.01, and p < 0.05, respectively), but there were no significant differences between the two groups on day 5 (p > 0.05, Fig 8E–8G). On day 1, the TNF-α and IL-10 levels in the LPS group were significantly higher than those in the control group (p < 0.05 and p < 0.01, respectively). On day 3, the TNF-α and HMGB1 levels in the LPS group were significantly higher than those in the control group (p < 0.01 and p < 0.05, respectively). On day 5, the expression levels of HMGB1 and IL-10 in the LPS-treated pigeons were higher than those in the saline-treated pigeons (p < 0.05 and p < 0.05, respectively; Fig 8H–8J).

## 4. Discussion

The integrity of the intestine plays an essential role in nutrient absorption, gut homeostasis, and animal growth and health [18,19,28]. VH, CD, and the VH/CD ratio are common indices for evaluating intestinal morphology [18]. The reason these indices are used is related to the fact that an increased VH and elevated VH/CD are directly correlated with increased epithelial turnover [30], and longer villi are linked with the activation of cell mitosis, while shortening of villi and deeper crypts lead to poor nutrient absorption [31], increased secretion in the gastrointestinal tract, and reduced performance [32]. A change in the integrity of the intestinal barrier induced by LPS can be detected by monitoring morphological changes in the intact intestinal villi and detecting chemical markers. Morphological changes in the intact intestinal villi are an indication of intestinal barrier injury, including LPS-induced injury [33,34]. D-LA is produced by intestinal microorganisms in vivo. When the intestinal mucosa is damaged and its permeability increases, D-LA will enter the blood, resulting in an increase in the serum D-LA level. The content of serum D-LA therefore reflects the health of the intestinal tract [35]. The present study demonstrated acute intestinal damage, with broken intestinal villi, a shortened VH, and an increased CD in the ileum, accompanied by higher D-LA levels in the serum.

It has been indicated that tight junctions between intestinal epithelial cells play an irreplaceable role in the function of the intestinal barrier [36]. Tight junctions act as receptors or targets of bacterial virulence factors during the infection of a range of viral and bacterial pathogens. Disruption of tight junctions leads to increased epithelial permeability and facilitates the translocation of pathogens into the body and colonization, usually resulting in diarrhea [37]. Increased expression of tight junction proteins enhances the function of the intestinal mucosal barrier. A series of proteins, including Claudins, Occludins, and ZOs, contribute to the formation of tight junctions. Numerous studies have reported that Occludin, Claudin3, and ZO-1 are key components of tight junction complexes and play important roles in intestinal permeability [38–40]. In the current study, the mRNA expression levels of Occludin, Claudin3, and ZO-1 were significantly decreased in the ileum of Wahui pigeons after LPS administration, suggesting that the intestinal barrier was damaged during injury.

Dysfunction of the intestinal barrier can increase intestinal mucosal permeability, induce the translocation of enteric pathogenic organisms, and in turn exacerbate the loss of intestinal barrier integrity. This can lead to systemic infection, multiorgan failure, and septic shock, which is a common pathological progression in many intestinal diseases [41]. A previous report showed that LPS exposure could lead to oxidative stress in the intestine [42]. LPS administration induces systemic and intestinal inflammation, accompanied by intestinal oxidative stress and increased intestinal permeability [4]. ROS are not only byproducts of inflammatory processes but also capable of causing damage to epithelial cell integrity and are closely linked to apoptosis in a variety of cell types [20]. Evidence from intestinal epithelial cells shows that elevated oxidative stress can inhibit cell proliferation and induce apoptosis [20,43]. In animals, there are many antioxidant enzymes and nonenzymatic antioxidants that protect the organism from oxidative damage [44]. MDA, as a product of lipid peroxidation, is acknowledged to be an index of cellular damage and excessive oxidative stress [45]. SOD converts superoxide (O_2_^−^) into H_2_O_2_, which is dissociated into H_2_O by CAT [46]. SOD levels indirectly reflect the capacity of organisms to neutralize ROS, and MDA content reflects the severity of the body’s exposure to free radicals [47]. In the current study, the levels of CAT and GSH were significantly decreased, but that of MDA was increased, indicating that oxidative stress occurred in the pigeons as a result of LPS treatment.

Oxidative stress and redox signaling can induce the release of proinflammatory factors, and the course of “ROS-induced inflammation” has been considered a contributor in the progression of most chronic diseases [48]. When foreign microorganisms invade, the innate immune system is activated to regulate antigen-presenting cells and initiate acquired immunity in the body. As an innate immune pattern recognition receptor, TLR4 can specifically recognize LPS from the outer membrane of gram-negative bacteria and forms the first line of defense called innate immunity. LPS binds to TLR4/MD2 to form a complex that selectively activates the MyD88-dependent pathway, resulting in the activation of the transcription factor NF-κB [49]. NF-κB plays important roles in the regulation of cytokines, adhesion molecules, inflammation, and oxidative stress and is also an important regulator of the intracellular inflammatory cascade. Numerous proinflammatory signals, including TNF-α and IFN-γ, promptly activate NF-κB to control target genes. Activated NF-κB enters the nucleus and stimulates the expression of a variety of genes that participate in immunoregulation and inflammatory reactions [50]. TNF-α is not only a factor related to the systemic inflammatory response but also the most important cytokine that causes an acute inflammatory response [51]. TNF-α is secreted mainly by macrophages, which activates macrophages to release a large number of proinflammatory factors, such as IL-1β, IL-6, and IFN-γ, through feedback regulation. This feedback plays a central role in the regulation of inflammation. TNF-α can also act as an endogenous pyrogen, causing fever and apoptosis [52,53]. HMGB1 is a fatal inflammatory factor caused by sepsis. Its release occurs later than that of inflammatory cytokines (such as IL-1β, IL-6, and TNF-α), and it is called a late risk signal factor [54]. HMGB1 activates *Aspergillus fumigatus* in alveolar macrophages in chronic obstructive pulmonary disease through the MyD88/NF-κB and syk/PI3K signal transduction pathways, leading to inflammation [55]. It has been reported that glycyrrhizic acid, an inhibitor of HMGB1, can mediate renal injury and inflammation in diabetic rats by regulating the activation of the RAGE/TLR4-related ERK and p38MAPK/NF-κB signaling pathways [56]. In addition, HMGB1 is overexpressed in thyroid cancer patient samples and cell lines and acts as a positive regulator of autophagy [57]. The stress in piglets responding to LPS challenge results in increased levels of proinflammatory cytokines [58,59]. In the present study, the mRNA expression levels of TNF-α, HMGB1, and IL-10 were increased in the ileum; the contents of TNF-α, HMGB1, and IL-10 were relatively high in the serum; and the protein levels of TNF-α and IL-10 were relatively high in the ileum. These findings suggest that LPS causes an inflammatory response could regulate by the TLR4/MyD88/NF-κB signaling pathway in Wahui pigeons.

It has been reported that a variety of Toll-like receptor ligands, such as double-stranded RNA and LPS, can lead to apoptosis [60–62]. As an important transcription factor, NF-κB not only controls inflammation but also acts in apoptosis [63]. NF-κB induces a variety of antiapoptotic factors, including inhibitors of caspase activation and activity, antiapoptotic Bcl-2 family members, and inhibitors of Jnk activation, which can prevent TNF-α-induced apoptosis [64]. Caspase-3 is another apoptosis-induced protein. Caspases generally play a central role in apoptosis induction [65]. Autophagy can be induced by a variety of stimuli and upstream signaling changes, including activation of the TLR4 signaling pathway. The pathogen-associated molecular molecule LPS activates the TLR4 signaling pathway and increases the level of autophagy in macrophages [66]. Another study demonstrated that LPS-induced autophagy is a TRIF-dependent and MyD88-independent signaling pathway [66]. Autophagy activated by TLR4 is regulated by the interaction of MyD88 and TRIF with Beclin-1 [67]. Binding of TLR4 with ligands induces the molecular interaction of MyD88, TRIF, and Beclin-1 but reduces the binding between Bcl-2 and Beclin-1, relieving the inhibition of Beclin-1 by Bcl-2 and promoting the occurrence of autophagy [67]. An NF-κB inhibitor, SN50, was found to inhibit NF-κB nuclear translocation and activate autophagy [68]. The key genes of autophagy and apoptosis pathway were almost continuously expressed at a high level after LPS challenge here, the intestinal injury probably took place along with autophagy and apoptosis of ileal cells.

## 5. Conclusion

In summary, continuous LPS challenge can induce oxidative stress, which promotes the release of inflammatory mediators, activates local and/or systemic inflammatory responses, causes apoptosis and autophagy, and reduces the expression of tight junction proteins, resulting in intestinal barrier dysfunction, thus reducing intestinal immunity and eventually leading to intestinal injury. This provides novel evidence for the pathological mechanism of intestine induced by *Salmonella* LPS in pigeon.

## Supporting information

S1 Table The primer sequences applied in real-time PCR. (DOCX)

S2 Table Morphological changes induced by LPS in the ileum of Wahui pigeons. (DOCX)
