# Supplementary material for: The influence on oxidative stress markers, inflammatory factors and intestinal injury-related molecules in Wahui pigeon induced by lipopolysaccharide
Source: PLoS One. 2021 May 12;16(5):e0251462. doi: 10.1371/journal.pone.0251462 (PMC8115843; doi:10.1371/journal.pone.0251462)
Supplement: S1 Table — (DOCX) [file pone.0251462.s001.docx]

**S1 Table. The primer sequences applied in real-time PCR**

| **Gene** | **Primer sequence (5'→3')** | **Product size (bp)** | **GenBank No.** |
| --- | --- | --- | --- |
| GAPDH | F: CCCAGAACATTATTCCAGC  R: CAGCACCCGCATCAAAG | 291 | NM_001282835.1 |
| Claudin3 | F: AGTGCAAGGTCTACGACTCC  R: GGCGGAGAGGATGAAGATGA | 200 | XM_005515008.2 |
| Occludin | F: TCTGCGGGTTCCTCATAGTC  R: TTCTTCACCCACTCCTCCAC | 154 | XM_005509325.2 |
| ZO-1 | F: GAGCTCACAAGCTACGCAAA  R: ACTTGTAGCACCATCTGCCT | 161 | XM_021299309.1 |
| Beclin1 | F: AGCTGGAGGACGTTGAGAAA  R: AGCTCCAGTTGCTGTCTCTT | 139 | XM_021280982.1 |
| Atg5 | F: GTCCAAGGTTTGTGGCTGTT  R: CAGAATGGGAACAGCACTGG | 188 | XM_005509471.2 |
| Bcl-2 | F: TACCTCCGAGACCCTGAGAA  R: CAGCAACAGGGAGAGAGGAA | 161 | XM_005509733.3 |
| Caspase-3 | F: CCTACCTGCCAGCAAGTCTA  R: CTTGCAGCATCTGCATCTGT | 159 | XM_005500178.2 |
| TLR4 | F: CTGAGCTCCGTGTTTGTCTG  R: CCCAGACCCAGATCCAGTTT | 127 | XM_005498384.2 |
| MyD88 | F: ACCAGTGGGTCTCATCAGTG  R: TGAGGCAGTGGCTGAAGTAA | 198 | XM_021296796.1 |
| NF-κB | F: TGTGTGTGCACAAAGCGTAA  R: AGACCGCATCGGCAATAGTA | 99 | XM_021294341.1 |
| HMGB1 | F: ATGCTCAGAACGGTGGAAGA  R: GGCCTCTTAGGTGCATTTGG | 162 | XM_013366679.2 |
| TNF-α | F: AGAGCTCGTCCGTTCTAAGG  R: GCCACAATCTGCTCCTGAAG | 188 | XM_005503259.2 |
| IL-10 | F: CTGCCTGCAAAGCTCAAAGA  R: TCATCTCCGACACAGACTGG | 154 | XM_021298374.1 |

**Note**: F, forward primer; R, reverse primer.
