# Supplementary material for: The influence on oxidative stress markers, inflammatory factors and intestinal injury-related molecules in Wahui pigeon induced by lipopolysaccharide
Source: PLoS One. 2021 May 12;16(5):e0251462. doi: 10.1371/journal.pone.0251462 (PMC8115843; doi:10.1371/journal.pone.0251462)
Supplement: S2 Table — (DOCX) [file pone.0251462.s002.docx]

**S2 Table. Morphological changes induced by LPS in the ileum of Wahui pigeons**

| **Parameter** | **Treatment** | **Day 1** | **Day 3** | **Day 5** |
| --- | --- | --- | --- | --- |
| VH (µm) | Control | 191.12 ± 35.18 | 280.75 ± 9.78 | 251.63 ± 4.91 |
|  | LPS | 140.84 ± 2.98* | 230.39 ± 13.08* | 171.95 ± 27.93** |
| CD (µm) | Control | 50.45 ± 2.21 | 42.66 ± 3.35 | 62.65 ± 8.16 |
|  | LPS | 51.49 ± 14.65 | 52.97 ± 11.45* | 64.19 ± 4.89 |
| VH/CD | Control | 4.04 ± 0.39 | 6.71 ± 0.002 | 3.93 ± 0.22 |
|  | LPS | 2.79 ± 0.06** | 4.41 ± 0.03** | 2.75 ± 0.13** |

**Note**: Compared with Wahui pigeons of the same age treated with saline. Data represent the mean ± standard deviation (n=6 in each group); VH: villus height, CD: crypt depth, VH/CD: ratio of villus height/crypt depth; *p < 0.05, **p < 0.01.
